# Carotid Occlusion Accentuates Aortic Stenosis and Cardiac Remodeling With Preserved Systolic Function in LDL Receptor-Deficient Mice

**DOI:** 10.3389/fphys.2020.578722

**Published:** 2021-01-11

**Authors:** Yandong Liu, Jiawei Cai, Lefeng Qu

**Affiliations:** Department of Vascular and Endovascular Surgery, Changzheng Hospital, Naval Medical University, Shanghai, China

**Keywords:** atherosclerosis, carotid stenosis, aortic stenosis, heart failure, ventricular remodeling

## Abstract

**Background:** Carotid atherosclerotic disease is associated with aortic stenosis and reduced cardiac function. The causality between carotid and cardiac pathologies is unknown. We aim to explore the effects of carotid stenosis or occlusion on cardiac pathology and function.

**Methods and Results:** We produced carotid obstruction or stenosis in 36 atherogenic mice with 150- or 300-μm tandem surgery or sham surgery. The structure and function of the heart were assessed by histology and animal ultrasound. The 150-μm group had larger plaque burden and thicker valve leaflets in the aortic root than did the control group. Also, the two surgery groups had a thicker left ventricular posterior wall and smaller internal diameter compared with controls. Increased myocardial fibrosis was also found in the 150-μm group compared with controls, although the surgery groups had preserved systolic function compared with that of controls.

**Conclusions:** In a mouse model, carotid occlusion accentuated the formation of aortic stenosis and promoted ventricular remodeling without impairing systolic function. Carotid atherosclerotic plaque may be a pathogenic factor for aortic stenosis and ventricular remodeling.

## Introduction

Carotid atherosclerotic disease is associated with cardiac disease. Hedberg et al. (2014) found that left ventricular systolic dysfunction was present significantly more often in patients with mild or severe carotid stenotic disease than in control subjects. Another study (Chahal et al., 2010) found that patients with multiple carotid plaques had decreased left ventricular ejection fractions, whereas patients who had only increased intima-media thickness did not, a result that may indicate that the severity of carotid stenosis affected cardiac function in a direct relationship. Among patients requiring aortic valve implantation, the prevalence of carotid plaque exceeded 90%, and carotid stenosis (≥50%) was present in more than 30% of patients (Steinvil et al., 2014). The cause–effect relationship between carotid stenosis and cardiac pathology is not known.

In this study, we evaluated the effect of carotid stenosis on aortic stenosis and cardiac function in mouse models of carotid stenosis.

## Materials and Methods

### Animal Models of Carotid Stenosis

Thirty-six seven-week-old male LDL receptor-deficient mice (Ldlr^–/–^; C57BL/6, Jackson Laboratory) were kept in a pathogen-free animal facility. The mice were fed a high cholesterol diet (0.4% fat + 1.1% cholesterol + 18.8% casein. Research Diets, America, D12104C) for 6 weeks. The mice were then randomized into three equal groups: 12 received 150-μm (outer diameter of the needle) tandem surgery (TS) on the right common carotid artery (simulating carotid occlusion); 12 received 300-μm (outer diameter of the needle) TS in the same location (simulating moderate-to-severe carotid stenosis); and 12 had sham operation, in which the common carotid artery was exposed without ligation. The TS surgery and anesthesia technique have been described (Chen et al., 2013). Briefly, the distal stenosis was located at the distal point 1 mm from the carotid artery bifurcation, and the proximal stenosis was located 3 mm from the distal stenosis. The stenoses were accomplished by placing a 6–0 surgical suture around the right common carotid artery together with a 150- or 300-μm needle that was tied to the artery and then removed. After the procedures, the high cholesterol diet was continued in all animals for 7 weeks until tissues were collected. All mice were cared for according to guidelines of the National Institutes of Health and Institutional Animal Care and Use Committee. All experiments with mice were approved by the Institutional Committee for the Use and Care of Laboratory Animals.

### Histology

At 20 weeks of age, mice were anesthetized with peritoneal injection of ketamine (100 mg/kg) and xylazine (10 mg/kg). Blood was collected from the inferior vena cava, and serum was prepared and stored at −80°C. Cold PBS (15 ml per mouse) was used for perfusion. The right common carotid artery, heart, and aorta were collected.

The degree of stenosis of the carotid artery and the plaque burden in the aortic root were assessed by measurement of the arterial lumen in hematoxylin–eosin (HE)-stained sections. Aortas were opened longitudinally with the intima exposed and stained with Oil Red O. Thickness of the aortic valve leaflet and cardiac fibrosis were evaluated in sections stained with Masson trichrome. The mean thickness of the aortic valve (μm) was determined by dividing the total area of the aortic valve through the total length of the valve. The maximal leaflet thickness (μm) was determined at the Arantius nodule in the middle of the thickest leaflet. Hearts were cut transversely at the level of the papillary muscle to visualize the ventricular myocardium. Quantitative morphometric analysis of cardiac fibrosis was conducted by measuring the percentage of blue area to the total area in three random fields (100 × magnification) for each sample. The area of plaque, fibrosis, and thickness of valve was quantified with Image J software (1.46r, United States).

### Analysis of Serum Inflammatory Cytokines and Lipids

Serum IL-6 and TNF-α levels were measured with ELISA kits (Invitrogen, Austria) according to the manufacturer’s instructions. Total cholesterol (TC), triglycerides (TG), low-density lipoprotein cholesterol (LDL-C), and high-density lipoprotein cholesterol (HDL-C) were enzymatically measured with the appropriate kits (Nanjing Jiancheng Biotechnology Company, China).

### Echocardiography

Mouse cardiac function was determined with a high-resolution ultrasonic imaging platform (Vevo2100). After being anesthetized with isoflurane, mice were placed supine on a heating pad, and respiratory rate, body temperature, and heart rate were monitored. The chests were shaved with depilatory cream. A heart rate of 300–550 beats per minute was maintained with mice under 0.5–1.5% isoflurane. One mouse in the 150-μm TS group had an uncontrollable heart rate, and it therefore was excluded for echocardiography analysis. Ejection fraction, cardiac output, fractional shortening, stroke volume, and volumes of the left ventricular were assessed with high-frequency probe MS-400 (30 MHz), using parasternal long-axis views; wall thicknesses and internal diameters were measured in parasternal short-axis M-Modes.

### Statistical Analysis

Data were expressed as mean ± standard error of the mean when normally distributed and otherwise with median (interquartile range). One-way ANOVA, two-way ANOVA, or Kruskal–Wallis test was used to determine the statistical significance of the differences among the three groups (Prism 5.0, GraphPad). Two-way ANOVA was applied to analyze the body weights in 6 and 13 weeks of age among three groups. A value of *p* < 0.05 was deemed statistically significant.

## Results

### Carotid Occlusion Accentuates Aortic Stenosis Independent of Altered Lipid Profiles and Inflammatory Status

**FIGURE 1:**
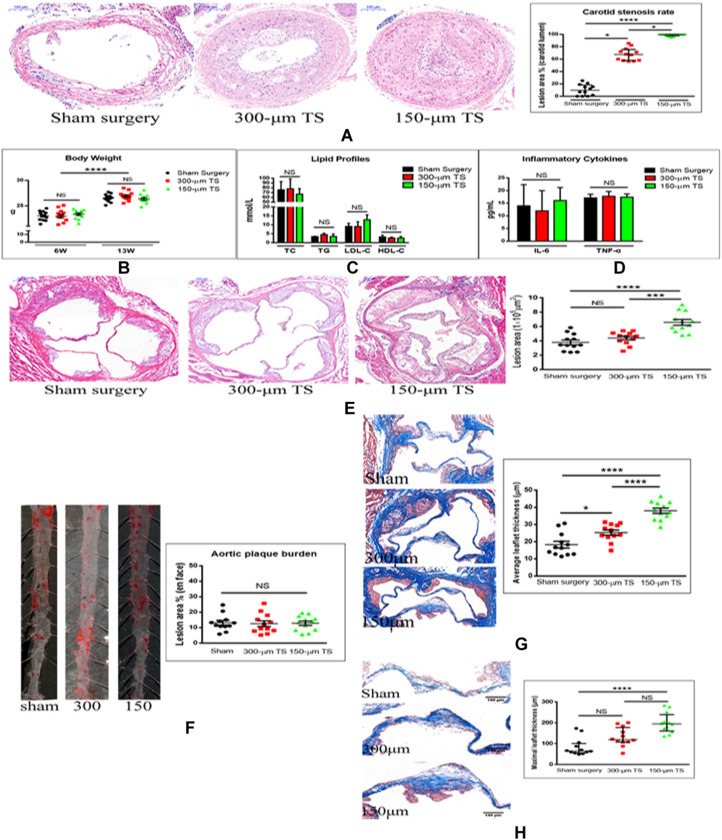
Carotid occlusion accentuated aortic stenosis. **(A)** Measurement of carotid stenosis in mice with 300-μm and 150-μm TS in hematoxylin–eosin (H-E)-stained sections. Scale bar = 100 μm. **(B)** Body weights of mice. **(C)** Lipid profiles of the three groups of mice. **(D)** Serum concentrations of inflammatory cytokines in three groups of mice. **(E)** Determination of lesion areas in aortic root of the three groups of mice measured in H-E-stained sections. Scale bar = 200 μm. **(F)** Quantification of plaque burden in thoracic and abdominal aorta in three groups of mice in Oil-O Red-stained sections. **(G)** Quantification of the average thickness of aortic valve leaflet in three groups of mice in Masson trichrome-stained sections. Scale bar = 100 μm. **(H)** Quantification of maximal thickness of aortic valve leaflet in three groups of mice in Masson trichrome sections. Scale bar = 100 μm. *n* = 12/groups, *p < 0.05, ***p < 0.001, and ****p < 0.0001. Black, red, and green columns or individual points represent sham surgery group, 300-μm TS group, and 150-μm TS group, respectively. TS, tandem surgery; TC, total cholesterol; TG, triglycerides; LDL-C, low-density lipoprotein cholesterol; HDL-C, high-density lipoprotein cholesterol; NS, not significant.

The TS-induced carotid stenosis model was constructed, and the degree of carotid stenosis was determined. Animals with 300-μm TS had a higher degree of stenosis in the carotid artery [67.5% (17.8)] than that in sham-operated animals [9.8% (17.7)] (*p* < 0.05), which simulated moderate-to-severe stenosis. Animals with 150-μm TS had a higher degree of carotid stenosis [99.8% (2.5)] than that in animals with 300-μm TS [67.5% (17.8)] (*p* < 0.05) and sham-operated animals [9.8% (17.7)], (*p* < 0.0001), which simulated carotid near-occlusion and occlusion (Figure 1A).

To determine whether the TS operations affected the general status of the mice, their body weights, serum lipid profiles (TC, TG, LDL-C, and HDL-C), and inflammatory cytokines (IL-6, TNF-α) were measured after 6 and 13 weeks of high-cholesterol diet; no differences in these variables were found among the three groups of animals (Figures 1B–D).

To determine whether carotid stenosis affected aortic stenosis, we measured plaque deposition in the aortic root and thickness of the aortic valve leaflets. Lesions in the aortic root were larger in the 150-μm TS group than in the 300-μm TS (*p* < 0.001) and sham groups (*p* < 0.0001), but lesion size in the 300-μm TS and sham groups was not significantly different (Figure 1E). Lesion size in the thoracic and abdominal aorta was not altered in the TS groups (Figure 1F), which indicates that carotid occlusion or stenosis did not affect atherosclerosis of arteries beyond the aortic valve. Next, the effect of carotid occlusion or stenosis on average and maximal valve leaflet thickness was analyzed. TS of 150 μm induced a significant increase in average leaflet thickness (*p* < 0.0001) and maximal leaflet thickness (*p* < 0.0001) compared with these thickness values in the sham-operated animals, and with average leaflet thickness in the 300-μm outer lumen diameter TS (*p* < 0.0001) animals. TS of 300 μm induced a significant increase in average leaflet thickness (*p* < 0.05) but no increase in maximal leaflet thickness compared to these thickness values in the sham group (Figures 1G,H). That the 150-μm TS had a greater effect on valve thickness than did the 300-μm TS is evidence that the severity of carotid stenosis affected aortic valve stenosis in a direct relationship.

### Carotid Occlusion Promotes Left Ventricular Remodeling Without Affecting Cardiac Function and Left Ventricular Volumes

**FIGURE 2:**
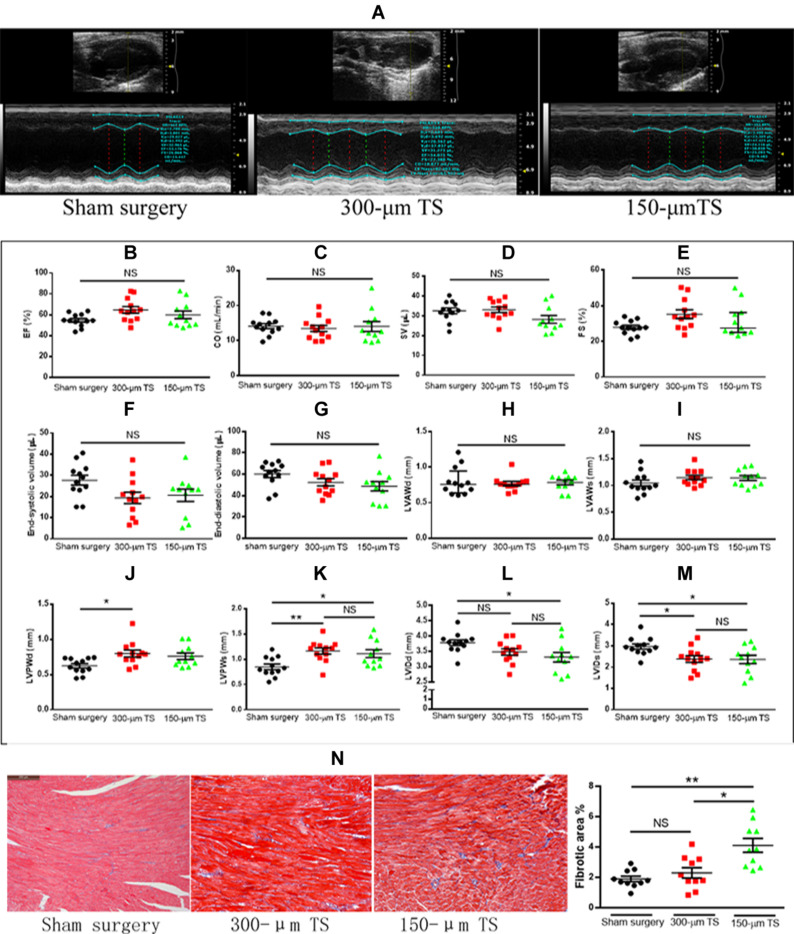
Carotid occlusion and stenosis promoted left ventricular remodeling. **(A)** Representative echocardiographic images of cardiac function and structure. **(B–E)** Cardiac function in three groups of mice. **(F,G)** Left ventricular volumes in three groups of mice. **(H–K)** The thickness of the left ventricular wall during diastole and systole. **(L,M)** Left ventricular diameter during diastole and systole. *n* = 11 or 12/groups (12 for control, 12 for the 300-μm TS group, and 11 for the 150-μm TS group), *p < 0.05, and **p < 0.01. **(N)** Quantification of cardiac fibrosis in three groups of mice in Masson trichrome sections. Scale bar = 200 μm. *n* = 10/groups, *p < 0.05, and **p < 0.01. Black, red, and green columns or individual points represent sham surgery group, 300-μm TS group, and 150-μm TS group, respectively. EF, ejection fraction; CO, cardiac output; SV, stroke volume; FS, fractional shortening; LVAWd, left ventricular anterior wall in diastole; LVAWs, left ventricular anterior wall in systole; LVPWd, left ventricular posterior wall in diastole; LVPWs, left ventricular posterior wall in systole; LVIDd, left ventricular internal diameter in diastole; LVIDs, left ventricular internal diameter in systole; NS, not significant.

A representative image of echocardiography for cardiac function and ventricular structure is illustrated in Figure 2A. The hemodynamic variables, including heart rate, respiratory rate, and cardiac output, are summarized in Table 1. The results were comparable between the three groups. There were no differences in ejection fraction, cardiac output, stroke volume, and fractional shortening among the three groups, indicating that carotid stenosis or occlusion did not cause overt cardiac failure (Figures 2B–E). The left ventricular volume and anterior-wall diameter was not significantly different among the three groups (Figures 2F–I). The 300-μm TS group developed a thicker left ventricular posterior wall than that of the sham group in diastole (*p* < 0.05) and systole (*p* < 0.01), whereas the 150-μm TS group developed a thicker left ventricular posterior wall than that in the sham group only in systole (*p* < 0.05) (Figures 2J,K). Moreover, the 150-μm TS group had a smaller left ventricular internal diameter than that of the sham group in diastole and systole (both *p* < 0.05), whereas the 300-μm TS group had a smaller internal diameter only in systole (*p* < 0.05) (Figures 2L,M). Furthermore, histological examination revealed that the 150-μm TS animals had larger fibrotic areas than those in the sham group (*p* < 0.01) and 300-μm TS group (*p* < 0.05), whereas no significant difference was observed in fibrotic areas between the 300-μm TS group and the sham group (Figure 2N). These results indicate that carotid stenosis or occlusion remodeled the left ventricle, characterized by thicker wall, decreased internal diameter, and increased myocardial fibrosis.

**TABLE 1:** The hemodynamic parameters of the three groups of mice.

Hemodynamic parameters	Sham surgery	300-μm TS	150-μm TS	*p*-value
Heart rate (bpm)	437.0 ± 15.3	414.8 ± 13.6	455.5 ± 8.1	0.093
Respiratory rate (counts/min)	30.0 (15.8)	28.0 (6.5)	24.0 (10.0)	0.162
Cardiac output (ml/min)	14.1 ± 0.7	13.5 ± 0.9	13.5 ± 1.4	0.139

## Discussion

Cardiac disease, including aortic stenosis and left ventricular remodeling, is strongly associated with carotid artery disease, but the cause–effect relationship between the two conditions is not known. To our knowledge, this is the first *in vivo* study of the effects of carotid stenosis or occlusion on aortic valve stenosis and cardiac function. In the LDL receptor-deficient mouse model, we found that plaque burden in the aortic root and thickness of aortic valve leaflets were significantly increased in response to carotid occlusive lesions, whereas indexes of systolic cardiac function were not significantly altered. Carotid occlusion and stenosis also resulted in increased thickness of the left ventricular wall and decreased internal diameter of the ventricle but did not induce overt heart failure. Nonetheless, results in our model suggest that carotid steno-occlusive disease caused at least incipient cardiac structural change, i.e., myocardial fibrosis and ventricular hypertrophy. The novel finding in this study is summarized as the following: carotid occlusion accentuates aortic stenosis, concentric hypertrophy, and myocardial fibrosis with preserved systolic function.

Our results are consistent with the observation that severe carotid disease in patients is correlated with abnormal heart conditions (Chahal et al., 2010; Hedberg et al., 2014). However, in variance with previous studies demonstrating that severe carotid disease is correlated with cardiac systolic dysfunction, carotid stenosis or occlusion in this study did not affect ejection fraction. However, the cardiac function was tested only 6 weeks postoperatively. Had we followed our animals longer, perhaps they would have developed overt heart failure. Our study also found that carotid occlusion could promote ventricular concentric hypertrophy. Similarly, an echocardiography-based clinical study demonstrated that more than half of the patients (53.1%) who had severe carotid stenosis had left ventricular concentric hypertrophy or remodeling (Duran Karaduman et al., 2020). Moreover, stenosis was more severe in the concentric hypertrophy group than in the group with normal left ventricular geometry. Another study also found that left ventricular myocardial remodeling occurred more frequently in hypertensive patients with severe carotid stenosis than in patients with moderate carotid stenosis (Korneva et al., 2006). Therefore, severe carotid stenosis or occlusion can at least lead to incipient cardiac structural change.

Severe carotid stenosis or occlusion can potentially lead to diastolic cardiac dysfunction, although that effect was not evident by echocardiography in this study. In this study, carotid stenosis or occlusion promoted concentric hypertrophy, and carotid occlusion promoted myocardial fibrosis. Concentric hypertrophy and myocardial fibrosis have been indicated as typical presentation of diastolic dysfunction (De Pergola et al., 2013; Lee et al., 2020). Carotid occlusion promoted both concentric hypertrophy and myocardial fibrosis, which indicated that carotid occlusion may be a pathogenic factor of diastolic cardiac dysfunction.

We feel that the mouse carotid stenosis model is useful for studying the effects of human carotid artery disease on cardiac function despite the species and methodological differences. In our model, we judged the severity of carotid stenosis through measurement of the degree of luminal stenosis in the cross-sectional angle, whereas in the previous human study, the severity of carotid disease was deduced from the enumeration of plaques (Chahal et al., 2010). We feel that our method is advantageous over that in the human study because, in clinical practice, the degree of carotid luminal stenosis is a determinant beyond that of plaque number for intervention of the stenosis (Brott et al., 2010); plaque enumeration may not reflect the severity of carotid disease and blood supply to the distal cerebral tissue.

Aortic stenosis shares risk factors with carotid atherosclerotic disease (Antonini-Canterin et al., 2009). In a prospective cohort study, the formation of carotid plaque was independently associated with incident aortic stenosis in a 20-year follow-up (Martinsson et al., 2014). Therefore, the presence of carotid atherosclerotic plaque could be a significant indicator for aortic stenosis. Additionally, our study further showed that plaque burden in the aortic valve could be aggravated by carotid stenosis, and aggravated more by carotid occlusion. These results imply that carotid plaque may be a promoter to aortic stenosis.

The findings that carotid occlusion in our model affected cardiac pathology independent of traditional risk factors, e.g., body weight, serum lipid values, and inflammatory status, imply that other pathophysiologic mechanisms are responsible. In a human study, revascularization of the occluded carotid artery may lead to a decrease in systolic blood pressure (Hasan et al., 2018), which implies that carotid occlusion causes increased blood pressure. High blood pressure might cause endothelial dysfunction and the development of atherosclerosis in the aortic root. However, our finding that the plaque burden in other peripheral arteries was not increased by TS surgery suggests that other mechanisms are responsible for this effect; one possibility is that the aortic stenosis and subclinical myocardial structural change are due to the increased afterload that accompanies carotid stenosis or occlusion. Detailed hemodynamic monitoring around the aortic root or ascending aorta in carotid stenosis is needed to resolve these issues.

The TS-induced model of carotid stenosis may be superior to previous models in exploring the effects of carotid atherosclerosis on the cardiac function. Previous models of carotid stenosis were mostly constructed by ligating the common carotid artery, without inducing the formation of carotid atherosclerotic plaque (Li et al., 2015; Gooch and Wilcock, 2016). Moreover, these models were constructed to simulate acute or chronic cerebral hypoperfusion, which may affect cardiac function. The TS model has advantages. First, the severity of carotid stenosis could be adjusted by adjusting the diameter of the needle, which facilitates exploring the effects of carotid stenosis or occlusion on cardiac function. Second, carotid atherosclerosis is a progressive disease, manifesting as early to advanced lesions. TS has induced gradual formation of plaque by affecting local hemodynamics (Chen et al., 2013). Third, carotid atherosclerotic plaques induced by TS are characterized by plaque rupture, thrombosis, and intra-plaque hemorrhage (Chen et al., 2013), which are also the main characteristics of advanced human plaque (Bentzon et al., 2014). Applying TS may simulate better the effects of carotid atherosclerosis on the cardiac function.

This study has limitations. First, the surgery was performed only on the right carotid artery, so the effects of right vs. left and bilateral carotid atherosclerotic disease were not examined. Second, the experiments were performed in the atherogenic model with no normal-animal control group. However, we feel that sham-operated animals were an appropriate control for the purposes of this study. Third, we did not study the long-term effects of carotid occlusion/stenosis on cardiac function or morphology. Fourth, diastolic function was not examined to ascertain whether heart failure with preserved ejection fraction formed in the 150-μm TS group. Finally, we did not explore mechanisms by which carotid occlusion leads to increased aortic stenosis and left ventricular remodeling; potential mechanisms responsible for this effect will be investigated in our future research.

In conclusion, carotid occlusion or stenosis in an LDL receptor-deficient mouse model promoted aortic stenosis and cardiac remodeling without impairing cardiac systolic function. These cardiac effects were not attributable to effects of the carotid artery occlusion/stenosis on body weight, serum lipid values, or inflammatory status. The murine model is useful for studying the pathophysiologic mechanisms responsible for aortic stenosis and left ventricular remodeling in patients with carotid atherosclerosis.

## Data Availability Statement

The raw data supporting the conclusions of this article will be made available by the authors, without undue reservation.

## Ethics Statement

The animal study was reviewed and approved by the Institutional Committee for the Use and Care of Laboratory Animals, Shanghai Jiao Tong University School of Medicine.

## Author Contributions

YL performed the animal experiments and wrote the manuscript. JC assisted in performing the animal experiments. LQ designed the experiments and revised the manuscript. All authors contributed to the article and approved the submitted version.

## Conflict of Interest

The authors declare that the research was conducted in the absence of any commercial or financial relationships that could be construed as a potential conflict of interest.
